# Clinicopathological features of small cell carcinoma of the uterine cervix in the surveillance, epidemiology, and end results database

**DOI:** 10.18632/oncotarget.16390

**Published:** 2017-03-21

**Authors:** Juan Zhou, San-Gang Wu, Jia-Yuan Sun, Li-Ying Tang, Huan-Xin Lin, Feng-Yan Li, Qiong-Hua Chen, Xin Jin, Zhen-Yu He

**Affiliations:** ^1^ Department of Obstetrics and Gynecology, The First Affiliated Hospital of Xiamen University, Xiamen, People's Republic of China; ^2^ Department of Radiation Oncology, Xiamen Cancer Hospital, The First Affiliated Hospital of Xiamen University, Xiamen, People's Republic of China; ^3^ Department of Radiation Oncology, Sun Yat-sen University Cancer Center, State Key Laboratory of Oncology in South China, Collaborative Innovation Center of Cancer Medicine, Guangzhou, People's Republic of China; ^4^ Eye Institute of Xiamen University, Fujian Provincial Key Laboratory of Ophthalmology and Visual Science, Medical College of Xiamen University, Xiamen, People's Republic of China; ^5^ Faculty of Basic Medicine, Medical College, Xiamen University, Xiamen, People's Republic of China

**Keywords:** cervical carcinoma, small cell, survival, prognosis

## Abstract

To investigate the clinicopathological characteristics and survival of small cell carcinoma of the cervix using Surveillance, Epidemiology, and End Results database. Patients with a diagnosis of small cell carcinoma of the cervix were included between 1988 and 2012. Kaplan-Meier method and Cox regression models were used. A total of 487 patients were included. Of the patients with known International Federation of Gynecology and Obstetrics stage and tumor grade, the stage IV disease was diagnosed in 37.9% patients, and 98.5% patients had poorly or undifferentiated histology. The 5-year cause specific survival and overall survival were 33.0% and 29.4%, respectively. In multivariate analysis, increasing age, advanced stage, and treatment by primary radiotherapy were associated with worse survival outcomes. Small cell carcinoma of the cervix is a rare disease with aggressive characteristics and prone to metastasize and is dismal in prognosis. Reduced survival was associated with increasing age, advanced stage, and treatment by primary radiotherapy.

## INTRODUCTION

According to the National Cancer Institute, there were approximately 12,900 new cases diagnosed and 4,100 deaths due to cervix cancer in 2015 [1]. About 90% of cervical cancer patients are squamous cell carcinoma. Small cell carcinoma of the cervix (SCCC) is a rare disease accounting for approximately 2–5% of uterine cervix malignancies [2–4].

SCCC was first reported by Reagan *et al*. in 1957 [5]. Like small cell carcinoma in other body sites, SCCC is highly invasive and prone to distant metastatic spread [3, 4, 6–8], causing poorer prognosis than other types of cervical cancer. The 5-year survival rates vary from 0% to 51% of patients with SCCC [8–11]. However, the clinicopathological features and biological behavior of SCCC in above studies were including limited number of patients. The aims of this study were to investigate the clinicopathological characteristics and survival in patients with SCCC using Surveillance, Epidemiology, and End Results (SEER) database.

## RESULTS

### Demographic, clinicopathological, and treatment characteristics

SEER database included 107,618 patients with small cell carcinoma and 65,761 patients with cervix cancer from 1988 to 2012. We included 487 patients with SCCC and a median age of 49 years (range, 19–95 years). The demographic, clinicopathological, and treatment characteristics are depicted in Table 1. The incidence of SCCC differed significantly according to age group. Patients with ages 40-49 years had a much higher incidence.

**Table 1:** Patient characteristics

Variable	n (%)
Age (years)	
Median (Range)	49 (19-95)
19-29	59 (12.1)
30-39	85 (17.5)
40-49	115 (23.6)
50-59	85 (17.5)
60-69	72 (14.8)
70-79	47 (9.7)
80+	24 (4.8)
Race	
Black	66 (13.6)
White	347 (71.3)
Other and unknown	74 (15.1)
Marital status	
No	212 (45.2)
Yes	257 (54.8)
Unknown	18
FIGO Stage	
IA	33 (7.2)
IB	82 (17.9)
I NOS	5 (1.1)
IIA	12 (2.6)
IIB	28 (6.1)
IIIA	10 (2.2)
IIIB	113 (24.7)
III NOS	1 (0.2)
IVA	9 (2.0)
IVB	163 (35.7)
IV NOS	1 (0.2)
Unknown	30
Grade	
Well differentiated	1 (0.3)
Moderately differentiated	4 (1.2)
Poorly/undifferentiated	326 (98.5)
Unknown	156
Tumor size	
Median size (cm) (range)	5.5 (0.2-21.0)
≤4 cm	95 (34.5)
>4 cm	180 (65.5)
Unknown	212
SEER stage	
Localized	112 (24.2)
Regional	180 (39.0)
Distant	170 (36.8)
Unknown	25
Local treatment modalities	
Hysterectomy ± radiotherapy	164 (44.4)
Radiotherapy	205 (55.6)
Unknown	118
Lymphadenectomy	
No	325 (68.6)
Yes	149 (31.4)
Node negative	71 (47.7)
Node positive	78 (52.3)
Unknown	13

Abbreviations: FIGO, International Federation of Gynecology and Obstetrics; SEER, Surveillance Epidemiology and End Results.

A total of 457 patients with International Federation of Gynecology and Obstetrics (FIGO) stage was available, 120 patients (26.3%) had stage I disease, 40 patients (8.8%) in FIGO stage II, 124 patients (27.1%) in stage III, and 173 patients (37.9%) in stage IV. Of the 331 patients with known histologic grade, 326 (98.5%) had tumors with poorly/undifferentiated grade. According to SEER stage (n = 462), 112 (24.2%) patients had localized stage, 180 (39.0%) had regional stage, and 170 (36.8%) had distant stage. A total of 275 patients with known tumor size, 180 (65.5%) had tumor size > 4 cm (Table 1).

Of the 369 patients with known local treatment modalities including hysterectomy and radiotherapy, 74 patients (20.1%) were treated with primary hysterectomy, 90 (24.4%) patients received hysterectomy and radiotherapy, and 205 (55.6%) patients received primary radiotherapy. A total of 149 patients received lymphadenectomy, and 78 (52.3%) of them were node-positive disease (Table 1).

### Survival

**Figure 1:**
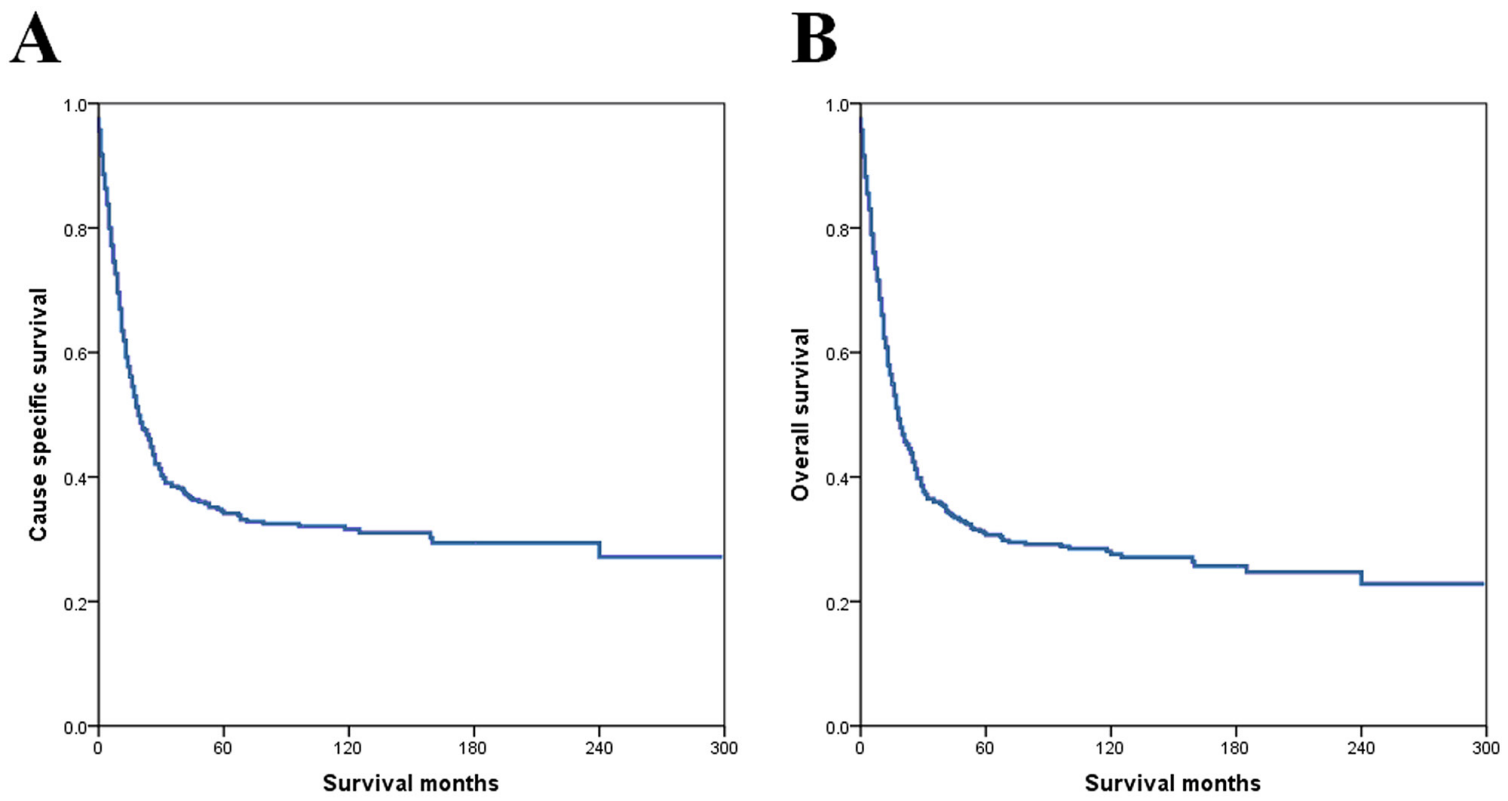
Cause-specific survival **(A)** and overall survival **(B)** for all patients.

Median survivals along with 5-year cause-specific survival (CSS) and overall survival (OS) for SCCC are summarized in Table 2. Mortality increased rapidly in the three years following diagnosis. Figure 1A shows the CSS of the SCCC patients. The 1-, 3-, 5-, and 10-year CSS was 61.6%, 37.3%, 33.0%, and 22.9%, respectively. The 1-, 3-, 5-, and 10-year OS was 60.4%, 34.7%, 29.4%, and 26.3%, respectively (Figure 1B). Patients with younger age, non-black racial status, married status, early stage, localized stage, node-negative tumors, and treatment with surgery ± radiotherapy had better CSS and OS (all *P* < 0.05). No differences in CSS and OS were observed among tumor grades and tumor size.

**Table 2:** Median, 5-year cause-specific survival and overall survival

Variable	CSS			OS		
	Median survival (months)	5-year	*P value*	Median survival (months)	5-year	*P value*
Age(years)						
19-29	60	50.2	< 0.001	57	48.3	< 0.001
30-39	29	40.6		29	40.6	
40-49	26	40.1		23	35.9	
50-59	19	32.9		18	26.4	
60-69	10	16.4		10	14.2	
70-79	9	16.0		9	9.0	
80+	5	12.5		5	12.5	
Race						
White	18	32.8	0.032	18	30.4	0.009
Black	13	23.2		11	16.9	
Other and unknown	29	42.0		27	36.5	
Marital status						
No	16	29.5	0.033	15	25.7	0.011
Yes	25	37.7		22	35.0	
FIGO stage						
I	240	60.7	< 0.001	125	55.2	< 0.001
II	29	40.3		29	40.3	
III	25	31.3		24	28.5	
IV	8	12.1		7	9.0	
Grade						
Well/ moderately differentiated	17	20.0	0.581	17	20.0	0.697
Poorly/undifferentiated	21	34.0		19	30.5	
Tumor size						
≤ 4 cm	31	37.7	0.101	26	32.7	0.128
> 4 cm	20	35.1		19	32.9	
SEER stage						
Localized	240	60.1	< 0.001	125	55.0	< 0.001
Regional	26	34.1		25	31.2	
Distant	7	12.6		7	9.3	
Nodal status						
Node negative	—	62.7	0.002	—	59.5	0.002
Node positive	27	38.5		26	35.6	
Local treatment modalities						
Hysterectomy ± radiotherapy	96	52.0	< 0.001	57	49.0	< 0.001
Radiotherapy	15	24.0		15	20.1	

Abbreviations: CSS, cause-specific survival; FIGO, International Federation of Gynecology and Obstetrics; OS, overall survival; SEER, Surveillance Epidemiology and End Results.

### Prognostic factors

Age at diagnosis, race, marital status, SEER stage, FIGO stage, lymph node status, and local treatment modalities were significant prognostic factors for CSS and OS by univariate analysis, whereas tumor size and tumor grade were not (Table 3).

**Table 3:** Univariate analysis of cause-specific survival and overall survival

Variable	CSS			OS		
	HR	95%CI	*P value*	HR	95%CI	*P value*
Age (years) (continuous variable)	1.024	1.017-1.031	< 0.001	1.026	1.019-1.033	< 0.001
Race						
White	1 [Reference]			1 [Reference]		
Black	1.351	0.982-1.858	0.064	1.477	1.095-1.993	0.011
Other	0.770	0.553-1.071	0.121	0.807	0.590-1.105	0.182
Marital status						
No	1 [Reference]			1 [Reference]		
Yes	0.781	0.619-0.984	0.036	0.752	0.601-0.940	0.012
FIGO stage						
I	1 [Reference]			1 [Reference]		
II	1.663	1.011-2.734	0.045	1.492	0.925-2.408	0.101
III	2.229	1.549-3.207	< 0.001	2.087	1.478-2.938	< 0.001
IV	5.522	3.927-7.764	< 0.001	5.213	3.781-7.187	< 0.001
Grade						
Well/ moderately differentiated	1 [Reference]			1 [Reference]		
Poorly/undifferentiated	0.760	0.282-2.047	0.588	0.824	0.306-2.218	0.702
SEER stage						
Localized	1 [Reference]			1 [Reference]		
Regional	1.999	1.414-2.827	< 0.001	1.961	1.413-2.722	< 0.001
Distant	5.324	3.767-7.523	< 0.001	5.153	3.707-7.163	< 0.001
Tumor size						
≤ 4 cm	1 [Reference]			1 [Reference]		
> 4 cm	1.312	0.944-1.823	0.106	1.272	0.929-1.740	0.133
Nodal status						
Node negative	1 [Reference]			1 [Reference]		
Node positive	2.066	1.278-3.342	0.003	2.050	1.288-3.262	0.002
Local treatment modalities						
Hysterectomy ± radiotherapy	1 [Reference]			1 [Reference]		
Radiotherapy	2.292	1.734-3.209	< 0.001	2.304	1.765-3.009	< 0.001

Abbreviations: CI, confidence interval; CSS, cause-specific survival; FIGO, International Federation of Gynecology and Obstetrics; HR, hazard ratio; OS, overall survival; SEER, Surveillance Epidemiology and End Results.

**Figure 2:**
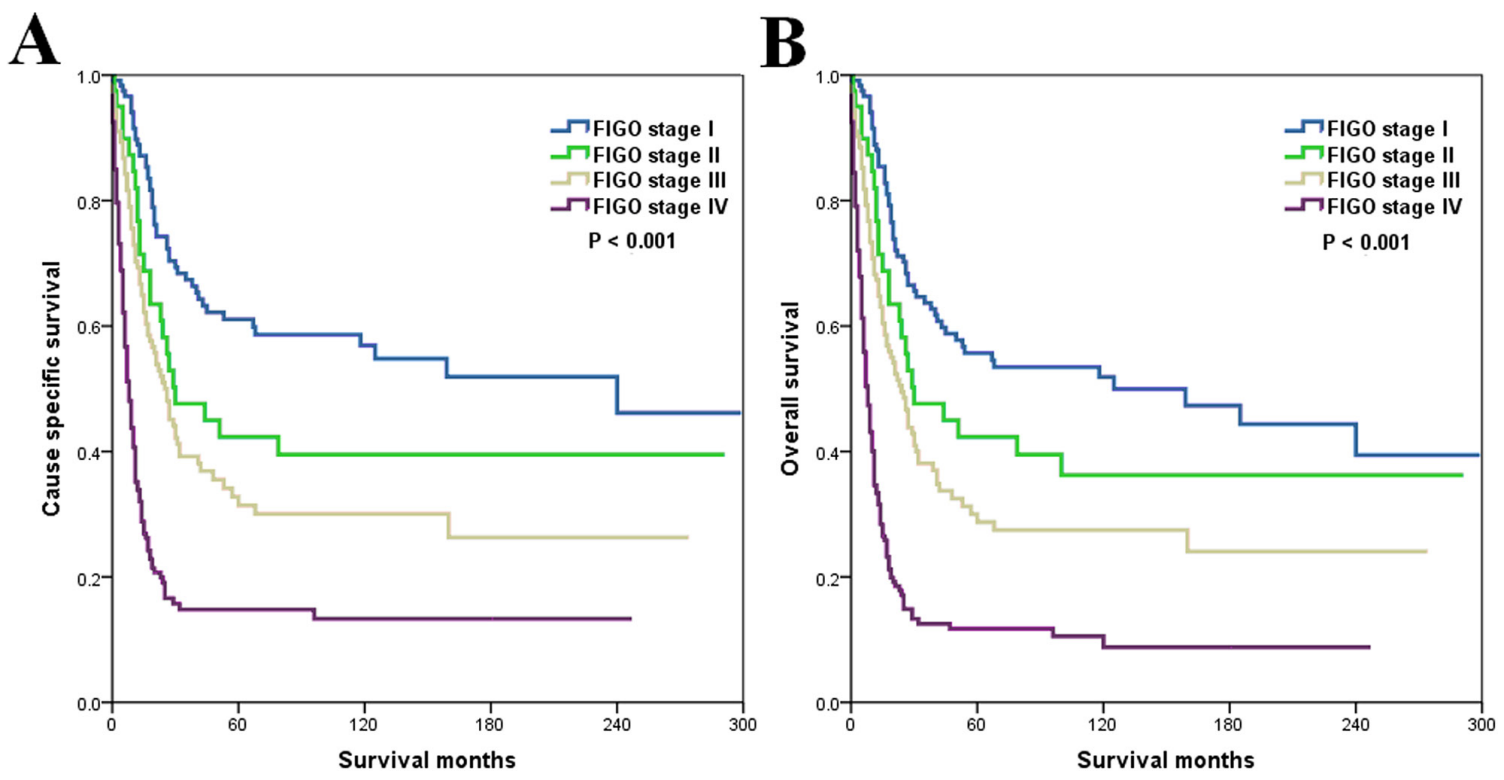
Cause-specific survival **(A)** and overall survival **(B)** according to International Federation of Gynaecology and Obstetrics stage.

A stepwise multivariable analysis of variables that were significant by univariate analysis showed that increasing age (CSS: hazard ratio [HR] 1.016, 95% confidence interval [CI] 1.008-1.025, *P* = 0.001; OS: HR 1.019, 95%CI 1.011-1.027, *P* < 0.001), advanced FIGO stage (CSS: HR 1.579, 95%CI 1.383-1.802, *P* < 0.001; OS: HR 1.545, 95%CI 1.362-1.752, *P* <0.001) and treatment by primary radiotherapy (CSS: HR 1.873, 95%CI 1.399-2.508, *P* < 0.001; OS: HR 1.854, 95%CI 1.402-2.452, *P* < 0.001) were significantly related to inferior CSS and OS (Table 4). Figure 2 shows CSS and OS according to FIGO stage.

**Table 4:** Multivariate analyses of cause-specific survival and overall survival

Variable	CSS			OS		
	HR	95%CI	*P value*	HR	95%CI	*P value*
**Entire group**						
Age (years) (continuous variable)	1.016	1.008-1.025	< 0.001	1.019	1.011-1.027	< 0.001
Race	—	—	—	0.953	0.794-1.143	0.602
Marital status	1.160	0.871-1.545	0.310	1.100	0.835-1.449	0.499
FIGO stage	1.579	1.383-1.802	< 0.001	1.545	1.362-1.752	< 0.001
SEER stage	0.817	0.542-1.620	0.817	0.951	0.564-1.605	0.852
Local treatment modalities	1.873	1.399-2.508	< 0.001	1.854	1.402-2.452	< 0.001
**With lymphadenectomy**						
Age	1.007	0.987-1.027	0.517	1.008	0.988-1.028	0.451
Race	—	—	—	1.178	0.836-1.659	0.348
Marital status	0.818	0.473-1.417	0.476	0.903	0.528-1.546	0.710
FIGO stage	1.365	1.060-1.757	0.016	1.316	1.030-1.680	0.028
SEER stage	0.548	0.100-2.992	0.487	0.475	0.088-2.579	0.389
Nodal status	0.911	0.237-3.497	0.892	0.886	0.232-3.378	0.859
Adjuvant radiotherapy	1.928	1.081-3.437	0.026	1.942	1.106-3.412	0.021

Abbreviations: CI, confidence interval; CSS, cause-specific survival; FIGO, International Federation of Gynecology and Obstetrics; HR, hazard ratio; OS, overall survival; SEER, Surveillance Epidemiology and End Results.

We further analyzed the prognostic factors for patients who underwent hysterectomy and lymphadenectomy. As adjusted for age, race, marital status, SEER stage, lymph node status, and local treatment strategies, advanced FIGO stage (CSS: HR 1.365, 95%CI 1.060-1.757, *P* = 0.016; OS: HR 1.316, 95%CI 1.030-1.680, *P* = 0.028) and local treatment modalities (hysterectomy and radiotherapy vs. hysterectomy) (CSS: HR 1.928, 95%CI 1.081-3.437, *P* = 0.026; OS: HR 1.942, 95%CI 1.106-3.412, *P* = 0.021) were still the independent prognostic factors of survival. The lymph node status has no effect on survival outcome in patients received lymphadenectomy.

## DISCUSSION

Most of the knowledge in the SCCC comes from single-institution reports with a limited numbers of patients [12–14]. Here, we identified 487 patients of histologically confirmed SCCC between 1988 and 2012 from the SEER registry. Previous studies have showed that SCCC accounts for 2–5% of all cervix cancers [2–4]. The incidence rate in our study was 0.7%, which is lower than previously reported. About 98.5% of SCCC patients have poorly or undifferentiated histology. Therefore, it is important to distinguish SCCC from poorly differentiated cervical cancer.

No prospective data was available to compare surgery with primary radiotherapy for resectable SCCC. Chen *et al*. previously reported a lower locoregional failure rate in patients who received primary radiotherapy than those who had primary surgery in stage I-II SCCC (6% vs. 27%, *P* = 0.009) [15]. Cohen *et al*. found that the OS (38.2% vs. 23.8%) was improved in patients who received radical hysterectomy (68.1% and 26.7% of patients underwent surgery in stage I-IIA and IIB-IVA diseases, respectively) [8]. In our previous study, we have found that radical surgery was the effective treatment for stage I-II SCCC [16]. In the present study, patients receiving surgery ± radiotherapy had better survival than those treated with primary radiotherapy. This difference may be partially explained that patients receiving surgery are usually diagnosed with stage I-II SCCC and those undergoing primary radiotherapy are often diagnosed as stage III-IV SCCC. Although surgery remains an important modality for early stage SCCC, chemotherapy-dominant comprehensive therapy is considered the main treatment for patients with advanced SCCC [3, 4].

In this study, FIGO stage was also significantly related to survival of SCCC patients. Several studies have found that patients with advanced stage were associated with poor survival [11, 17–19]. A total of 37.9% of patients had stage IV disease at the initial diagnosis in this study. In other studies, 4.6–23.5% of patients had stage IV SCCC at the initial diagnosis [6, 11, 20]. The discrepancy in the proportion of patients with stage IV SCCC might be ascribed to the sample size. A worse survival outcome was also associated with increasing age on multivariate analysis. In patients with non-specific pathological type of cervical cancer, the poor prognoses associated with increasing age have been reported in our previous studies [21, 22]. However, age had no significant prognostic value in other studies with SCCC subtype [8, 9, 11, 23].

It has been reported that the incidence of lymph nodes metastasis vary from 39.4% to 70% of SCCC patients who receive lymph node resection [9, 11, 23], which was significantly higher than that in patients with squamous cell carcinoma and adenocarcinoma of the cervix [24, 25]. However, the prognostic role of nodal status in SCCC remains controversial. We found that 52.3% of SCCC patients had node-positive, and univariate analysis indicated that patients with positive lymph nodes had a poorer prognosis. However, multivariate analysis failed to identify lymph node status as a prognostic factor. Our findings were consistent with two retrospective studies by Liao *et al*. (n = 293) [23] and Wang *et al*. (n = 179) [11] with a relatively large number of patients. Therefore, more studies are warranted in the future to assess the prognostic value of lymph node status in SCCC patients.

The treatment modalities of SCCC and small-cell lung cancer are similar due to similar biological behavior including lymph node involvement, vascular invasion, and early recurrence [26–28]. Chemotherapy is an important component of multimodality therapy. Several studies have found that concurrent chemotherapy or adjuvant chemotherapy improved survival in SCCC [7, 8, 15, 29, 30]. In this study, we cannot analyze the effect of chemotherapy on the survival of SCCC patients due to the limitations of SEER database. The British Columbia Cancer Agency began using multimodality regimen including platinum-based chemotherapy in the treatment of SCCC in 1989 [7]. Several studies also showed that most of patients received multimodality therapeutic strategies including chemotherapy regimens after 1990 [11, 17, 29, 30]. We included patients between 1988 and 2012, who were in a chemotherapy-based treatment era, and the survival rates of different FIGO stage in our study were similar to those of previous studies [11, 17, 29, 30]. The SEER-Medicare database contains the chemotherapy data. However, only patients with 65 years or older were included in the SEER-Medicare database, which will significantly reduce the sample size and limit the generalizability of the study (101 patients older than 65 years in the present study).

The pathological factors including surgery margin, lymphovascular invasion and parametrial invasion are the prognostic factors of cervical cancer. SEER program also lacks the information of the above pathological factors. The prognostic value of above pathological factors in SCCC remains controversial. A study be Wang *et al*. showed that positive surgical margins was an adverse prognostic factor for failure-free survival (*P* < 0.001) but not in cancer-specific survival (*P* = 0.593), lymphovascular invasion and parametrial extension had no effect of survival in multivariate analyses [11]. However, the other two studies did not report the prognostic value of surgical margin on survival [9, 15]. Lymphovascular invasion and parametrial involvement were also found to have no prognostic value in SCCC [8, 15, 23]. Therefore, there were different prognostic factors in SCCC compared to squamous cell carcinoma and adenocarcinoma of the uterine cervix.

There are several limitations of this study. The first is the inherent biases existing in any retrospective study. However, the major strength of the present study is the ability to describe the epidemiology, clinicopathological features, treatment trends, and survival outcomes of this rare disease using a population-based study. Second, SEER database lack of information about centralized pathologic review, pathological factors (margin status, lymphovascular invasion, and parametrial invasion), details of radiation therapy and chemotherapy, and the data of local and distant recurrence. In addition, there is little information available to guide the choice of treatment in certain patients. Although prospective studies have a greater scientific impact than retrospective studies, prospective data on the outcome of different local treatment modalities in SCCC was not available.

In conclusion, SCCC is a rare disease with aggressive characteristics and prone to metastasize and is dismal in prognosis. Reduced survival was associated with increasing age, advanced stage, and treatment by primary radiotherapy. More studies are needed to confirm our results and develop optimal management of SCCC.

## MATERIALS AND METHODS

### Patients

Data were obtained using SEER*Stat software (Version 8.2.1; available at http://www.seer.cancer.gov/seerstat) from the current SEER database, which is maintained by the National Cancer Institute and consists of 18 population-based cancer registries. We included SCCC patients from 1988 to 2012 and permission to access research data files was obtained [31]. The tumors were classified based on their primary site of presentation using the International Classification of Disease for Oncology, Third Edition. Data released from the SEER program did not require informed patient consent and this study was approved by the Ethics Committee of the First Affiliated Hospital of Xiamen University and the Sun Yat-Sen University Cancer Center.

### Demographic and clinicopathological variables

The following demographic and clinicopathological variables were collected from the SEER program: age, race, marital status, tumor grade, tumor size, FIGO stage, SEER stage, lymph node status, and local treatment modalities. Vital status and underlying cause of death were also were recorded.

### Statistical analysis

The chi-square test and Fisher exact probability tests were used to evaluate the differences between qualitative data. Univariate and multivariate Cox proportional hazards model were used to identify prognostic factors predictive of CSS and OS. Multivariable analyses were used to identify independent prognostic factors that were significantly related to CSS and OS in univariate analyses. CSS and OS were calculated using the Kaplan-Meier method and compared using the log-rank test. The SPSS statistical software package, version 21.0 (IBM Corporation, Armonk, NY, USA) was used for statistical analysis. A value of *P* < 0.05 was considered to be statistically significant.
